# The flavonoid beverage Haelan 951 induces growth arrest and apoptosis in pancreatic carcinoma cell lines in vitro

**DOI:** 10.1186/s12906-015-0734-0

**Published:** 2015-07-03

**Authors:** Juliane Rothe, Michael Wakileh, Katrin Dreißiger, Heike Weber

**Affiliations:** Institute of Clinical Chemistry and Laboratory Medicine, University of Rostock, Ernst-Heydemann-Straße 6, 18057 Rostock, Germany

## Abstract

**Background:**

A major challenge in pancreatic cancer treatment is the resistance of human pancreatic cancer cells to apoptosis. Soy isoflavones and calpain inhibition have been suggested to exert inhibitory effects on cancer development and progression. We investigated the effects of the isoflavone containing beverage Haelan 951 and the calpain inhibitor PD150606 on the viability, growth and apoptosis of the human pancreatic cancer cell lines CAPAN-1 and BxPC-3, on the rat pancreatic cancer cell line AR42J, and on human fibroblasts as the control cell line.

**Methods:**

Cellular viability and proliferation were determined using the LDH cytotoxicity and WST-1 assay, respectively. Apoptosis was detected by flow cytometric analyses of Annexin V-FITC labeled-cells, TUNEL assay and caspase activation. Student’s *t* test or Mann–Whitney Rank Sum test were used to compare the data.

**Results:**

Haelan concentrations lower than 8 % showed no cytotoxic effects, whereas higher concentrations led to necrosis. Eight percent Haelan induced significant growth inhibition of CAPAN-1 and BxPC-3 cell lines by 30 % and 35 %, respectively, compared with the control. The proliferation rate of AR42J cells decreased by 50 %, whereas the fibroblasts remained unaffected. An 1.1-fold increase in apoptosis was found in CAPAN-1 cells, whereas the number of apoptotic BxPC-3 cells was elevated 2-fold. The number of apoptotic AR42J cells and fibroblasts was elevated 1.5-fold, each. Inhibition of calpain activity amplified the Haelan-induced growth inhibition of CAPAN-1 and BxPC-3 cells, but failed to amplify the growth inhibition of Haelan-treated AR42J cells. In fibroblasts, calpain inhibition induced Haelan-independent growth inhibition. Calpain inhibition also amplified the Haelan-induced apoptotic activity in all cancer cell lines, but exerted no further effect in fibroblasts.

**Conclusions:**

The proliferation-inhibiting and apoptosis-inducing effects of Haelan are highly dependent on cell type and concentration administered. The results show for the first time that Haelan may be a promising candidate in the treatment of human pancreatic cancer, and its anticancer activity may be potentiated when administered with calpain inhibitors.

## Background

The most common type of pancreatic cancer (PC) is the highly aggressive adenocarcinoma originating from the exocrine pancreas. PC is the fourth most common cause of cancer-related mortality in the US and Europe with a 5-year survival of just 4 % [1, 2]. The high mortality and dismal survival rate both strongly suggest that the evaluation of therapeutic agents is urgently needed. A major challenge in the treatment of PC has been the lack of protective responses to various chemotherapies, which has been attributed to the resistance of PC cells to apoptosis [3]. Thus, increasing the sensitivity of tumor cells to apoptosis may be a promising strategy for the development of efficient chemotherapies that extend survival.

Apoptosis is defined as a programmed form of cell death induced to eliminate genetically altered cells without causing severe host reaction. Apoptosis can be induced by various extracellular and intracellular stimuli leading to the activation of three main pathways, the extrinsic (death receptor-mediated), the intrinsic (mitochondrial) and the endoplasmic reticulum stress-mediated pathway. An increasing number of studies suggests that naturally occurring compounds may be suitable candidates for cancer treatment by inducing apoptosis, such as bufalin, a component of the Chinese herbal medicine Chan-Su, the 4-herb Chinese medicine formulation PHY906, the traditional Chinese medicine herbal mixture LQ, and [6]-gingerol, a ginger phytochemical [4–7]. Isoflavones, a subclass of naturally occurring and biologically active polyphenolic phyto-estrogens, have also been shown to possess anticancer activities. They deregulate cell cycle progression, induce apoptosis, function as antioxidants, modulate multiple cell signalling pathways and inhibit tumor invasion [8]. Isoflavones are found in plant-derived foods and beverages such as vegetables, fruits, green tea and wine [9, 10]. A very rich source of isoflavones is the soybean, containing the predominant glycoside compounds genistin and daidzin along with other glycosides [10]. Fermentation of soy hydrolyzes the glycosides to form isoflavone aglycones such as genistein, daidzein and others that are absorbed faster and in greater amounts than their glucosides upon oral administration in humans [11].

Several studies on a variety of cancer cell lines suggest that a mixture of isoflavones is more effective in suppressing cancer growth than the isolated compounds alone [12–15]. Thus, in the present study, we investigated the anticancer effect of the commercially available fermented soy beverage, Haelan 951 (Hael), mainly contains genistein, genistin, and daidzein using the human PC cell lines, BxPC-3 and CAPAN-1, the rat PC cell line, AR42J, and human fibroblasts as control to detect cytotoxic activity to non-cancer cells [16]. A further aim was to investigate whether inhibition of calpain may amplify Hael-induced anticancer activity. Calpains are a family of cytosolic neutral cysteine proteases that are strictly controlled by the cytosolic Ca^2+^ concentration and the endogenous inhibitor protein, calpastatin. Calpain, in particular the ubiquitous isoforms μ- and m-calpain, has been identified to be involved in cancer development and progression, including cell transformation, migration and tumor invasion, apoptosis/survival, as well as angiogenesis via signal-dependent limited cleavage of its substrates [17, 18].

Our results show for the first time that Hael may be a promising agent in the treatment of human pancreatic cancer, leading to growth arrest and apoptosis without cytotoxic effects. Inhibition of the calpain activity was found to improve the therapeutic efficacy of Hael.

## Methods

### Reagents

Haelan 951 Platinium Formula (Hael) (batch no. 4050901) was purchased from Haelan Products, Inc. Woodinville, WA, U.S.A.). The beverage contains 555 μM diadzein and 955 μM genistein [16]. Cell proliferation reagent WST-1 and the TUNEL (terminal deoxynucleotyl transferase-mediated dUTP nick end labelling) assay were purchased from Roche (Mannhein, Germany). Penicillin and streptomycin were obtained from Biochrom Seromed (Berlin, Germany). 3-(4-Iodophenyl)-2-mercapto-(Z)-2-propenoic acid (PD150606) and 1,2-Bis-(o-aminophenoxy)-ethane-N,N,N',N'-tetraacetoxymethylester (BAPTA-AM) were purchased from Calbiochem-Novabiochem (Nottingham, UK). Accutase was obtained from Millipore Corp. (Temecola, CA, U.S.A.). FBS (heat inactivated) and all other cell culture material were obtained from Life Technologies (Paisley, UK). The CytoGlo Annexin V-FITC Apoptosis Detection Kit was purchased from IMGENEX (San Diego, CA, U.S.A).

### Cell cultures

The human pancreatic carcinoma cell lines BxPC-3 and CAPAN-1, the rat pancreatic carcinoma cell line AR42J and the human skin fibroblast cell line were obtained from the American Type Culture Collection (Rockville, MD, U.S.A.) The AR42J cells and the fibroblasts were grown in DMEM supplemented with 10 % (v/v) FCS. The CAPAN-1cell line was grown in IMDM supplemented with 10 % (v/v) FBS. The BxPC-3 cell line was cultured in RPMI containing 10 % (v/v) FBS. All culture media were supplemented with 50 U/ml penicillin and 50 μg/ml streptomycin. The cells were grown at 37 °C in a 5 % CO_2_ humidified atmosphere. Growing cells were checked for viability and confluence by light microscopy. The cells were detached by accutase according to the manufacturer’s recommendation.

### Exposure of PC cell lines and fibroblasts to increasing concentrations of Hael

The three PC cell lines and human fibroblasts (pro well: 4 x 10^4^ cells / 2 ml cell culture medium) were added to 24-well plates including the control and blank. After reaching subconfluence, the medium was removed and the cells were incubated with increasing concentrations of Hael (2 % - 32 %) containing in 2 ml of the corresponding cell culture medium for 24 and 48 h. The control cells were incubated with medium only.

### Exposure of PC cell lines and fibroblasts to 8 % Hael in combination with calpain inhibitors

The three PC cell lines and human fibroblasts (4 x 10^4^ cells/2 ml cell culture medium) were incubated with 8 % Hael in the presence or absence of the calpain inhibitor PD150606 (20 μM) or the Ca^2+^ chelator BAPTA-AM (10 μM) for 24 h. The inhibitors were dissolved in DMSO for a final concentration of 1 %. Control and Hael-treated cells without inhibitor were incubated with vehicle.

### Determination of cellular viability

After termination of the experiment, cell membrane damage was assessed by measuring the release of lactate dehydrogenase (LDH) into the incubation medium using the LDH test kit and Synchron LX 20 analyzer from Beckman Coulter (Krefeld, Germany).

### Cell proliferation assay

To characterize cellular proliferation rate, the WST-1 assay was used. The WST-1 assay is based on the cleavage of the tetrazolium salt WST-1 in viable cells only, leading to the formation of a soluble formazan salt, which can then be measured photometrically. After termination of the cell culture experiment, the cells were washed twice with PBS, and 50 μl WST-1 in 500 μl cell culture medium was added. After 2 h, the absorbance was measured at 450/600 nm using a Benchmark Plus microplate reader (BioRad, Munich, Germany).

### Detection of apoptotic cells by the in situ cell death detection kit, POD

Apoptosis leads to cleavage of genomic DNA. For apoptosis detection, these strand breaks can be identified by labeling free 3’-OH termini with modified nucleotides in an enzymatic reaction using the in situ cell death detection kit, POD. Cells were cultured in culture slides (BD Biosciences, Bedford, MA, U.S.A.) and incubated with or without 8 % Hael for 24 h. Thereafter, they were fixed with fixation solution for 2 min on ice. In the next step, 50 μl TUNEL-POD was added. The chamber was closed and incubated for 30 min at 37 °C. After three washing steps with PBS, 50 μl substrate solution was added which was followed by a 10 min incubation period at RT. Thereafter, the cells were visualized under a transmission light microscope.

### Quantification of apoptosis using the CytoGlo Annexin V-FITC Apoptosis Detection Kit

Cells were harvested, pelleted by centrifugation and washed twice with PBS. Aliquots of 1 x 10^6^ cells were suspended in 100 μl binding buffer (10 mM HEPES, 135 mM NaCl, 5 mM CaCl_2_) followed by the addition of 5 μl staining reagent. After incubation in the dark at RT for 20 min, 400 μl binding buffer was added, and the cells were analyzed using a flow cytometer (Becton Dickinson, Heidelberg, Germany, excitation: 488 nm; emission: 530 nm; software version 1.2). Ten thousand events were measured per sample.

### Detection of apoptosis by SR-FLICA multicaspase assay

Apoptosis was detected using the sulforhodamine FLICA (Fluorescent Labeled Inhibitors of Caspases) apoptosis detection kit containing sulforhodamyl-L-valylalanylaspartyl fluoromethyl ketone (SR-VAD-FMK). The FLICA reagent is cell permeable and non-toxic. It reacts covalently with activated caspases and is retained in apoptotic cells, while unbound reagent will diffuse out of the cell.

Aliquots of 10^5^ cells were seeded onto chamber slides and grown in their respective cell culture media at 37 °C for 24 h. Then, cells were incubated with 8 % Hael for 24 h followed by application of FLICA solution according to the manufacturer’s recommendation. After 1 h incubation at 37 °C in 5 % CO_2_ environment, the medium was removed and DAPI (1.5 μl per 300 μl medium) was added followed by an additional 5 min incubation period. Finally, the cells were observed under a fluorescence microscope Nikon Eclipse E600 using a band pass filter (excitation 510–560 nm, emission 590 nm for red fluorescence) and an UV-filter (excitation 340–380 nm, emission 435–485 nm for DAPI stain). The intensity of the red fluorescence correlates with the concentration of activated caspases.

### Statistical methods

Data are expressed as means + SEM. Mean values of normally distributed data with equal variance were compared by Student`s *t* test. If the normality test failed, the data were compared by Mann–Whitney Rank Sum test. The statistical software package SigmaStat 3.5 from Jandel Corporation (Erkrath, Germany) was used. P ≤ 0.05 was considered as statistically significant.

## Results

### Effects of different Hael concentrations on the viability of PC cell lines and fibroblasts

**Fig. 1:**
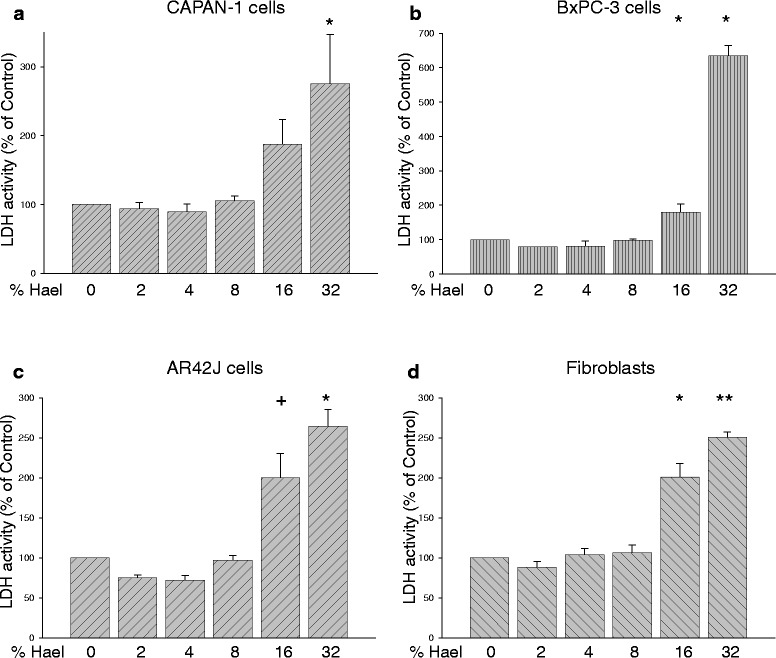
Dose-dependent effect of Haelan on the viability of pancreatic carcinoma cells and fibroblasts. CAPAN-1 cells (**a**), BxPC-3 cells (**b**), AR42J cells (**c**) and fibroblasts (**d**) were incubated with or without increasing concentrations of Haelan. After 24 h, cellular viability was assessed using the LDH cytotoxicity assay. Haelan concentrations of 2 %-8 % induce no cytotoxic effects, whereas concentrations higher than 8 % cause significant plasma membrane damage in all cell lines investigated. The results are expressed as mean + SEM (n = 3-12). ^+^P < 0.1, *P < 0.05, **P < 0.01, control vs. Haelan-treated cells

The human PC cell lines CAPAN-1 and BxPC-3, the rat PC cell line AR42J and human fibroblasts were incubated with Hael at concentrations ranging from 2 % to 32 % for 24 h (Fig. 1a-d). Cellular viability was determined by measuring the LDH release into the incubation medium. At concentrations of 2 %, 4 % and 8 %, Hael induced no cytotoxic effects in any of the cell lines investigated. However, at a concentration of 16 % Hael, significant damage of the plasma membrane was observed, leading to an increase in the LDH release by 87 % in CAPAN-1 cells, 80 % in BxPC-3 cells (P < 0.05), and by 100 % each in AR42J cells (P < 0.1) and fibroblasts (P < 0.05), versus corresponding control. In response to 32 % Hael, a further dramatic increase in cell damage was recorded in all cell lines investigated, particularly in the BxPC-3 cells.

### Effects of different Hael concentrations on the proliferation of PC cell lines and fibroblasts

**Fig. 2:**
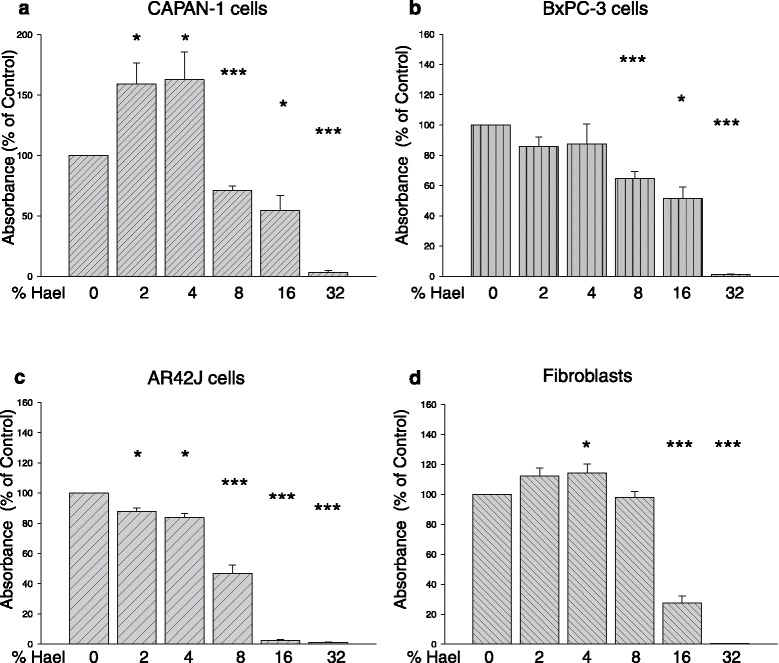
Effect of Haelan on the proliferation of pancreatic carcinoma cells and fibroblasts. CAPAN-1 cells (**a**), BxPC-3 cells (**b**), AR42J cells (**c**) and fibroblasts (**d**) were incubated with or without increasing concentrations of Haelan. After 24 h, the proliferation was measured using the WST-1 assay. Haelan exerts significant effects on cell proliferation dependent upon cell type and concentration. The concentration of 8 % Haelan induces growth arrest in all pancreatic cancer cells, but not in the fibroblasts. The results are expressed as mean + SEM (n = 3-12). *P < 0.05, ***P < 0.001, control vs. Haelan-treated cells

To investigate the effect of Hael on cellular proliferation, the activity of mitochondrial dehydrogenases was measured using the WST-1 assay (Fig. 2a-d). Concentrations of 2 % and 4 % Hael induced a significant increase in the proliferation of CAPAN-1 cells by approximately 60 % in comparison with the control (P < 0.05). A small increase in proliferation was also observed on the fibroblasts using these concentrations. In AR42J cells, however, concentrations of 2 % and 4 % Hael significantly reduced the proliferation rate. The proliferation rate of BxPC-3 cells at 2 % and 4 % Hael tended to be reduced, although the results were non-significant.

Exposure to 8 % Hael significantly reduced the proliferation rate of all PC cell lines but not of fibroblasts. In particular, the proliferation of CAPAN-1 and BxPC-3 cells was decreased by 30 % (P < 0.001) and 35 % (P < 0.001), respectively. The AR42J cell line displayed a decrease in proliferation rate by 50 % (P < 0.001) when treated with 8 % Hael. Treatment with 16 % or 32 % Hael induced strong cytotoxic effects in all cell lines investigated. Based on these results, a concentration of 8 % Hael was chosen for further experiments.

### Effect of 8 % Hael on the proliferation of PC cell lines and fibroblasts at different incubation times

**Fig. 3:**
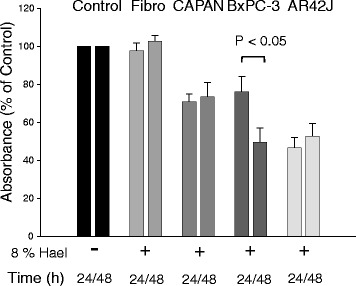
Time-dependent effect of Haelan on the proliferation of pancreatic carcinoma cells and fibroblasts. CAPAN-1 cells, BxPC-3 cells, AR42J cells and fibroblasts were incubated with or without 8 % Haelan. After 24 h and 48 h, cell proliferation was measured using the WST-1 assay. Haelan induces a reduction in the proliferation of the pancreatic cancer cells, but not of the fibroblasts after 24 h. The proliferation of the cancer cells shows no reduction after 48 h with the exception of BxPC-3 cells. The results are expressed as mean + SEM (n = 6-12)

Incubating the cell lines with 8 % Hael for 48 h caused no further decrease in the proliferation rate of CAPAN-1 cells, AR42J cells and fibroblasts when compared to the proliferation rate measured after 24 h (Fig. 3). However, the proliferation of BxPC-3 cells was reduced by 35 % (P < 0.05) at 48 h versus 24 h incubation.

### Effect of 8 % Hael on apoptosis in PC cells and fibroblasts

**Fig. 4:**
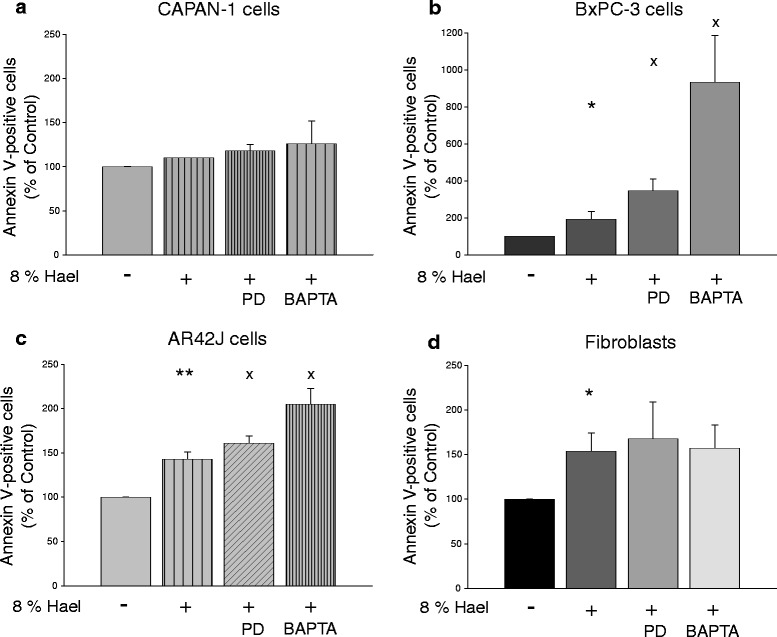
Haelan-induced apoptosis in pancreatic carcinoma cells and fibroblasts. CAPAN-1 cells **a**, BxPC-3 cells **b**, AR42J cells **c** and fibroblasts **d** were incubated with 8 % Haelan or vehicle in the presence or absence of the calpain inhibitor PD150606 or Ca^2+^ chelator BAPTA-AM and stained after 24 h using the CytoGlo Annexin V-FITC Apoptosis Detection kit. The percentages of Annexin V-positive cells are assessed by flow cytometric analyses. Haelan induces apoptosis in all cell types investigated. PD150606 or BAPTA-AM increases the degree of Haelan-induced apoptosis in all cells with the exception of the fibroblasts. The results are expressed as mean + SEM (n = 3-4). *P < 0.05, **P < 0.01, control vs. Haelan-treated cells, ^x^P < 0.05, Haelan-treated cells vs. cells treated with Haelan in the presence of PD150606 or BAPTA-AM

To examine whether Hael causes apoptosis, cells were stained with Annexin V-FITC and flow cytometric analyses were undertaken after 24 h. In CAPAN-1 cells, 8 % Hael increased the number of Annexin V-positive cells 1.1-fold, and in BxPC-3 cells 2.1-fold (P < 0.05) compared to the corresponding control (Fig. 4a, b). The number of apoptotic AR42J cells and fibroblasts was increased by a factor of 1.5 each (P < 0.01 and P < 0.05), respectively, (Fig. 4c, d).

**Fig. 5:**
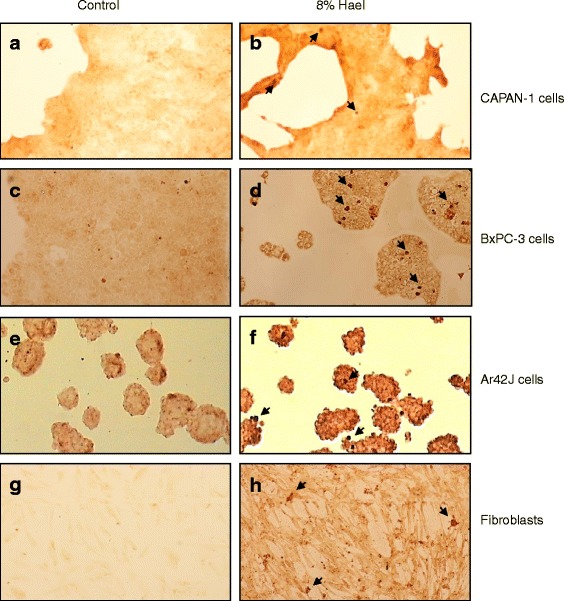
Haelan-induced apoptosis in pancreatic carcinoma cell lines and fibroblasts. CAPAN-1 cells (**a**, **b**), BxPC-3 cells (**c**, **d**), AR42J cells (**e**, **f**) and fibroblasts (**g**, **h**) were grown on culture slides and treated with or without 8 % Haelan for 24 h. For apoptosis detection, the TUNEL assay was used. The light micrographs demonstrate that Haelan causes apoptosis in all cell types investigated when compared with the respective controls. (n = 2; original magnification x10; arrows indicate apoptotic cells)

Using the TUNEL POD assay for apoptosis detection, the results also revealed that 8 % Hael induced apoptosis in all cell lines investigated. Thus, in the CAPAN-1 cell population, only a small number of apoptotic cells (stained dark brown) was visible compared with the control, whereas in all other cell lines, the number of apoptotic cells was of greater degree (Fig. 5a-h).

**Fig. 6:**
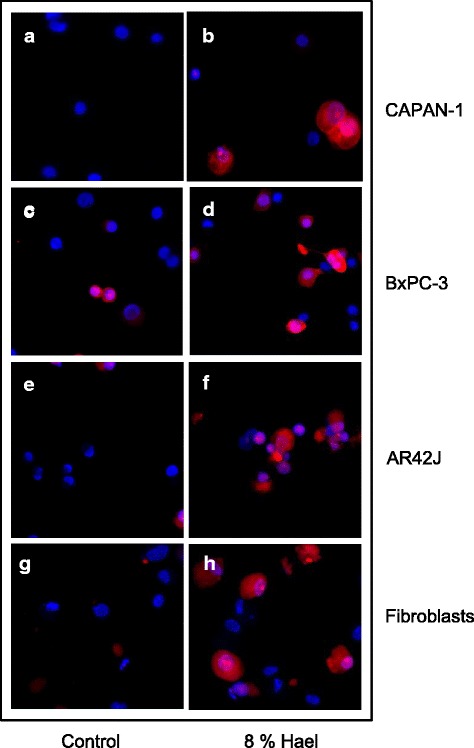
Haelan-induced apoptosis in pancreatic carcinoma cells and fibroblasts. CAPAN-1 cells **a**, **b**, BxPC-3 cells **c**, **d**, AR42J cells **e**, **f** and fibroblasts **g**, **h** were incubated with or without 8 % Haelan and dually stained with sulforhodamine-labeled caspase inhibitor, SR-VAD-FMK, and DAPI. The representative micrographs demonstrate only few red-colored apoptotic cells in the control groups and a much greater number of red-colored apoptotic cells in the Haelan-treated groups when analyzed after 24 h (n = 2); (original magnification x 10)

To investigate whether caspase activation may be involved in Hael-induced apoptosis, cells were dually stained with SR-VAD-FMK reagent and DAPI, and viewed through a fluorescence microscope. Cells exhibiting a very bright red staining patter were undergoing apoptosis in a caspase-dependent manner, whereas cells appearing faint red were just in early apoptotic stage. Compared with the corresponding control groups, a greater number of red-colored cells was found in the Hael-treated CAPAN-1 cells (compare Fig. 6a, b), in Hael-treated BxPC-3 cells (compare Fig. 6c, d), in Hael-treated AR42J cells (compare Fig. 6e, f) and in Hael-treated fibroblasts (compare Fig. 6g, h).

### Effect of calpain inhibition and cytosolic Ca^2+^ binding on cellular viability and growth inhibition of Hael-treated PC cells and fibroblasts

**Fig. 7:**
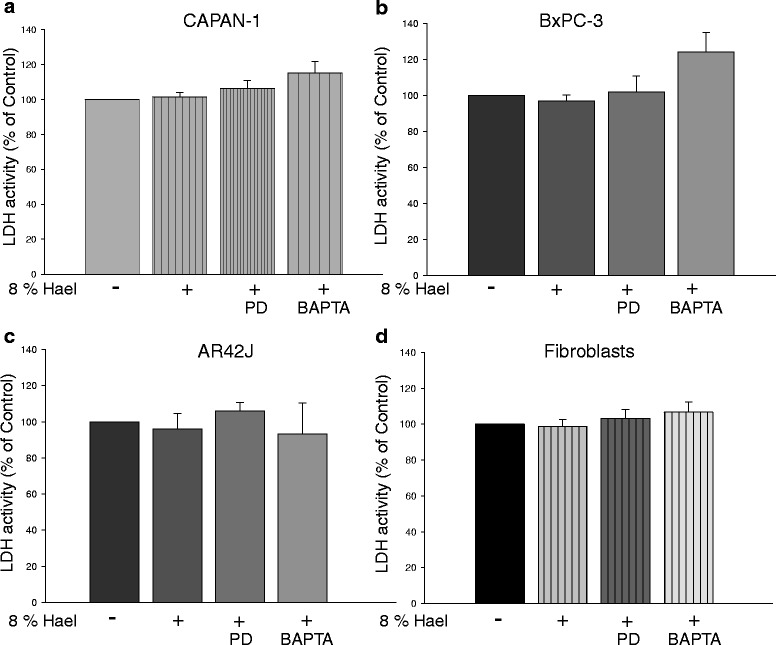
Effect of calpain inhibition and cytosolic Ca^2+^ binding on the viability of Haelan-treated pancreatic carcinoma cells and fibroblasts. CAPAN-1 cells **a**, BxPC-3 cells **b**, AR42J cells **c** and fibroblasts **d** were incubated with 8 % Haelan or vehicle in the presence or absence of the calpain inhibitor PD150606 or the Ca^2+^ chelator BAPTA-AM. After 24 h, the cellular viability was assessed using the LDH cytotoxicity assay. Inhibition of calpain activity or Ca^2+^ chelation has no effect on the viability of Haelan-treated pancreatic carcinoma cells and fibroblasts. The results are expressed as mean + SEM (n = 3-7)

We first tested whether treatment of cell lines with the specific calpain inhibitor PD150606 or the Ca^2+^ chelator BAPTA-AM in combination with 8 % Hael lead to cellular damage. As assessed by the LDH cytotoxicity assay, no cytotoxic effects were found when the cells were incubated with Hael in the presence of PD150606 in comparison with Hael treatment alone (Fig. 7a-d). Exposure to Hael in the presence of BAPTA-AM increased the LDH release of CAPAN-1 and BxPC-3 cells but not of AR42J cells and fibroblasts.

**Fig. 8:**
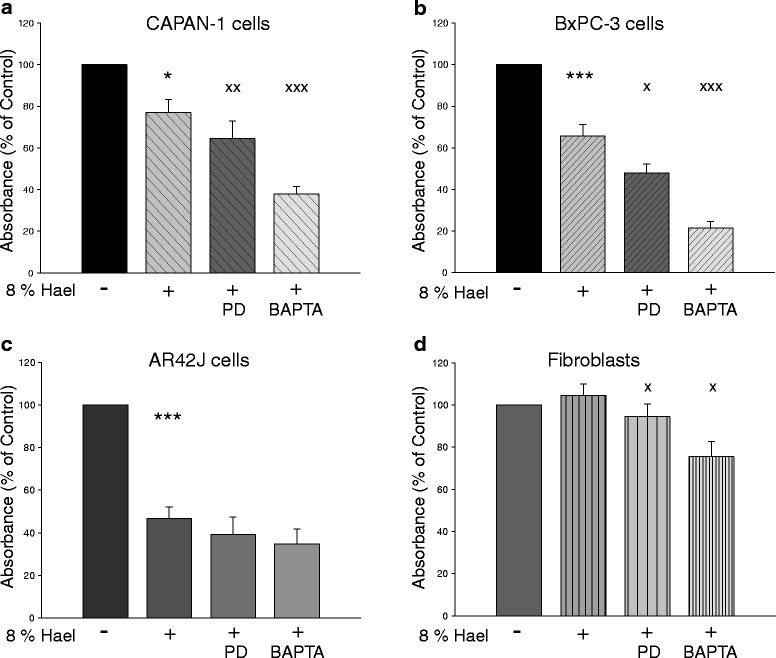
Effect of calpain inhibition and cytosolic Ca^2+^ binding on the proliferation of Haelan-treated carcinoma cells and fibroblasts. CAPAN-1 cells (**a**), BxPC-3 cells (**b**), AR42J cells (**c**) and fibroblasts (**d**) were incubated with 8 % Haelan or vehicle in the presence or absence of the calpain inhibitor PD150606 or the Ca^2+^ chelator BAPTA-AM. After 24 h, the proliferation was measured using the WST-1 assay. Inhibition of calpain activity or Ca^2+^ chelation induces an additional decrease in the proliferation of Haelan-treated CAPAN-1 and BxPC3 cells, but not in the AR42J cells. In fibroblasts, Haelan fails to induce growth arrest, whereas calpain inhibition or binding of Ca^2+^ initiates this mechanism. The results are expressed as mean + SEM (n = 6-12). *P < 0.05, ***P < 0.001, control vs. Haelan-treated cells, ^x^P < 0.05, ^xx^P < 0.01, ^xxx^P < 0.001, Haelan-treated cells vs. cells treated with Haelan in the presence of PD150606 or BAPTA-AM

Next, we investigated the effect of PD150606 or BAPTA-AM on cell proliferation. Compared to Hael treatment alone, co-incubation of CAPAN-1 and BxPC-3 cells with Hael and PD150606 caused an additional reduction in the proliferation by 15 % (P < 0.01) and 25 % (P < 0.05), respectively (Fig. 8a, b). No further reduction in Hael-induced growth inhibition was observed in AR42J cells (Fig. 8c). Interestingly, whereas Hael exhibited no inhibitory effect on the proliferation rate of fibroblasts, a small but significant decrease (P < 0.05) was observed under combined treatment with PD150606 (Fig. 8d).

Cytosolic Ca^2+^ binding by BAPTA-AM also affected cell proliferation. BAPTA-AM inhibited Hael-induced proliferation rate in CAPAN-1 cells by 50 % (P < 0.001), and in BxPC-3 cells by 70 % (P < 0.001), (Fig. 8a, b). In AR42J cells, BAPTA-AM failed to further decrease Hael-induced growth inhibition, but caused a Hael-independent growth inhibition in fibroblasts by approximately 30 % (P < 0.01) compared to Hael treatment alone (Fig. 8c, d).

### Effect of calpain inhibition and cytosolic Ca^2+^ binding on Hael-induced apoptosis in PC cell lines and fibroblasts

Co-treatment of Hael with PD150606 or BAPTA-AM increased the number of apoptotic CAPAN-1 cells 1.2-fold and 1.3-fold, respectively, when compared to Hael-treatment alone (Fig. 4a). A stronger amplification of Hael-induced apoptosis was achieved in the other PC cell lines. Thus, in BxPC-3 cells, treatment of Hael together with PD150606 or BAPTA-AM increased the number of apoptotic cells 1.9-fold (P < 0.05) and 4.8-fold (P < 0.05), whereas in AR42J cells, an additional increase by 1.1-fold (P < 0.05) and 1.4-fold (P < 0.05) was observed, respectively, (Fig. 4b, c). In fibroblasts, however, neither PD150606 nor BAPTA-AM amplified the degree of Hael-induced apoptosis (Fig. 4d).

## Discussion

In this study, we investigated the effect of the fermented soy-based beverage Hael 951 on cell viability, proliferation and apoptosis in human and rat PC cell lines and human fibroblasts. Hael 951 is rich in soybean proteins, flavonoids, selenium, zinc, and vitamin A, B_2_, B_12_, C, D_3_, E and K. In addition, it contains essential fatty acids such as linolenic and linoleic acids [19]. Our results suggest that the effect of Hael strongly depends on concentration and cell type. Thus, we found that lower concentrations of 2 % and 4 % Hael stimulated the proliferation of human CAPAN-1 cells and fibroblasts within the 24 h observation period but not the proliferation of AR42J and BxPC-3 cells. The stimulating effect may be due to genistein and daidzein, which are two of the major components of Hael. Both compounds have been reported to increase the proliferation of prostate epithelial and estrogen-sensitive human breast cancer (MCF-7) cells, but not of estrogen-insensitive human breast cancer (MDA-MB-231) cells [20–22]. In MCF-7 cells, this effect has been attributed to an increase in DNA synthesis [23]. CAPAN-1 cells and fibroblasts are estrogen receptor-expressing cells as well [24, 25]. Consequently, Hael-induced increase in the proliferation rate of these cell lines may also be explained with an increase in DNA synthesis. In human BxPC-3 and rat AR42J cells, the low Hael concentrations of 2 % and 4 % were found to induce a small growth inhibition.

In response to higher (8 %) concentrations of Hael, the inhibitory effect on cellular proliferation was magnified, leading to significant growth arrest in all cancer cell lines investigated. The proliferation of fibroblasts was not affected by 8 % Hael. In support of these findings, the corresponding LDH activities measured in parallel were unchanged, suggesting that the decrease in the WST-1 levels was not caused by cell membrane damage, but reflects genuine growth inhibition. The finding that the proliferation of BxPC-3 cells was further reduced after 48 h, whereas a similar effect was not found in the other cancer cell lines may indicate that BxPC-3 cells were of higher sensitivity to Hael.

Hael concentrations higher than 8 %, such as 16 % and 32 %, dramatically reduced the WST-1 levels in the cancer cell lines as well as in fibroblasts. These findings were accompanied by increased corresponding LDH releases, suggesting that higher Hael concentrations do not induce growth arrest but exert strong cytotoxic effects. Similarly, high concentrations of the major Hael component genistein have been reported to cause damage to various cell types, including the human ovarian cancer cell line SK-OV-3, and primary rat cortical neurons [12, 26, 27]. Genistein was also shown to dramatically reduce the number of the pancreatic CAPAN-1, PANC-1 and MIAPaCa-2 cells [28]. Another major Hael component, daidzein, caused damage to HeptG2 cells and primary rat cortical neurons when administered in high concentrations [27, 29]. Based on these results, further experiments were performed with 8 % Hael. This concentration is approximately 100-fold higher than that found in the plasma samples obtained from a patient 1 h after ingesting 4-oz of Hael [16].

Investigating the apoptosis-inducing activity, the results show that 8 % Hael induces apoptosis in all PC cells and fibroblasts, in which the CAPAN- 1 cells showed the lowest sensitivity to 8 % Hael, and the BxPC-3 cells the highest sensitivity. In addition, the results also reveal that the cells entered the apoptotic pathway in a caspase-dependent manner.

Concerning the molecular mechanisms underlying Hael-induced growth arrest and apoptosis, several explanations can be offered. Flavonoids including genistein and daidzein have been shown to induce DNA damage via inhibition of tyrosine kinases and topoisomerase-II, which in turn may lead to activation of p53 tumor-suppressor protein-dependent pathways [30, 31]. Low levels of p53 induced in response to low or repairable stress have been observed to result in cell cycle arrest leading to expression of survival genes that are able to repair any cell damage [32]. The precise point within the cell cycle the flavonoids block seems to be dependent on their concentration, the exposure time, the experimental conditions, and the cell line investigated [33]. Irreparable DNA damage leads to higher p53 expression that initiates apoptosis to prevent the propagation of genetic defects to successive generations of cells [32]. The extent of p53-dependent gene expression has been identified to be cell line-specific as well [34].

In further experiments, we show that inhibition of the calpain activity improves the therapeutic efficacy of Hael. Using the highly specific cell membrane permeable calpain inhibitor PD150606, which does not block other proteases, the results show a significant amplification of Hael-induced growth inhibition in CAPAN-1 and BxPC-3 cells and a downward trend in the AR42J cell proliferation rate. Interestingly, Hael alone failed to induce growth arrest in fibroblasts as already mentioned above, but simultaneous inhibition of calpain led to a small inhibitory effect on proliferation. A variety of studies have shown that p53 is a calpain substrate [35]. Consequently, inhibition of calpain activity may lead to growth arrest by increasing the availability of p53 [36]. Furthermore, calpain has been shown to regulate the cell cycle at multiple points [18]. In cancer cells, the protease cleaves inhibitors of cyclin-dependent kinases p21/Cip1 and p27/Kip1, which in turn facilitates cell proliferation [17, 37]. Therefore, inhibition of calpain activity may lead to growth arrest.

Investigating the effect of calpain inhibition on Hael-induced apoptotic activity, our results reveal an increase in Annexin V-positive cells in all PC cell populations investigated. Western blot analyses of active caspase-3 confirmed these findings (data not shown.) In support of our data, inhibition of the calpain activity has been observed to initiate apoptosis in a variety of cancer cell lines, including human prostate tumor cells, hepatocellular tumor cells (SK-HEP-1, HLF), colorectal cancer cells (RKO, DLD-1), Jurkat and Molt cells [36, 38–40]. There is growing evidence suggesting that calpain cleaves upstream caspases including caspase-3, −8 and −9 leading to inactivation of apoptosis [36, 40, 41]. Consequently, inhibition of calpain activity should lead to activation of apoptosis as observed in the present study. In Hael-treated fibroblasts, however, calpain inhibition failed to enhance apoptosis, suggesting that the underlying molecular mechanisms are different between non-cancer and cancer cells.

The observation that BAPTA-AM amplified the Hael-induced growth inhibition in all PC cell lines and provoked growth inhibition in Hael-treated fibroblasts indicates that Ca^2+^-dependent mechanisms may contribute to the regulation of cancer cell and non-cancer cell proliferation alike. BAPTA-AM-induced cytosolic Ca^2+^ binding also increased the apoptotic activity of Hael in CAPAN-1, BxPC-3 and AR42J cells, but failed to enhance Hael-induced apoptosis in fibroblasts. S100 proteins, which are a large family of Ca^2+^ binding proteins of the EF-hand type, may be involved in these observations. S100 proteins have been reported to play a role in the regulation of numerous processes, including proliferation, differentiation, apoptosis, Ca^2+^ homeostasis, energy metabolism, inflammation and migration/invasion [42]. Upon Ca^2+^ binding, most S100 proteins undergo a conformational change that enables them to interact with their target proteins [43]. Thus, binding of cytosolic Ca^2+^ by BAPTA-AM may prevent the activation of S100 proteins, which consequently may lead to an amplification of Hael-induced growth arrest and apoptosis. In support of our assumption, RNAi-mediated knockdown of S100A4 has been reported to block cell growth and motility, and to induce apoptosis in the human pancreatic cancer cell lines BxPC-3, PANC-1 and MIA PaCa-2 via activation of pro-apoptotic signaling proteins including caspase-3, and caspase-9 [44, 45]. Similarly, in cardiac fibroblasts, knockdown of S100A4 has been found to significantly suppress cell proliferation via increasing p53 expression, whereas the effect on apoptosis has not been investigated [46].

In summary, our results demonstrate that 8 % Hael exerted no cytotoxic effects on all PC cells investigated as well as on fibroblasts. In addition, the results show that 8 % Hael led to growth inhibition in the PC cells, which was amplified by the inhibition of calpain activity or by cytosolic Ca^2+^ binding with the exception of the AR42J cells. In fibroblasts, Hael failed to induce growth arrest, whereas calpain inhibition and cytosolic Ca^2+^ binding triggered this mechanism. Hael was also found to cause apoptosis in the PC cell lines, which was further amplified when the cells were treated with Hael in combination with calpain inhibitor or Ca^2+^ chelator. Hael also induced apoptosis in fibroblasts, but both inhibitors had no apoptosis amplifying effect.

## Conclusions

Although the precise mechanisms underlying Hael-induced growth arrest and apoptosis in the presence or absence of calpain inhibitor or Ca^2+^ binding compounds have to be elucidated, the results suggest that these agents may be suitable targets for the treatment of human PC.

## References

[1] Hariharan D, Saied A, Kocher HM (2008). Analysis of mortality rates for pancreatic cancer across the world. HPB (Oxford).

[2] Jemal A, Siegel R, Ward E, Hao Y, Xu J, Murray T (2008). Cancer statistics, 2008. CA Cancer J Clin.

[3] Yang CS, Maliakal P, Meng X (2002). Inhibition of carcinogenesis by tea. Annu Rev Pharmacol Toxicol.

[4] Li M, Yu X, Guo H, Sun L, Wang A, Liu Q (2014). Bufalin exerts antitumor effects by inducing cell cycle arrest and triggering apoptosis in pancreatic cancer cells. Tumour Biol.

[5] Saif MW (2008). Is there a role for herbal medicine in the treatment of pancreatic cancer?. JOP.

[6] Zhang L, Wu C, Zhang Y, Liu F, Zhao M, Bouvet M (2013). Efficacy comparison of traditional Chinese medicine LQ versus gemcitabine in a mouse model of pancreatic cancer. J Cell Biochem.

[7] Kim SO, Kim MR. [6]-Gingerol prevents disassembly of cell junctions and activities of MMPs in invasive human pancreas cancer cells through ERK/NF- κ B/Snail signal transduction pathway. Evid Based Complement Alternat Med. 2013 (2013), Article ID 761852, 9 p. http://dx.doi.org/10.1155/2013/761852. 10.1155/2013/761852 PMC3800596 24204396

[8] Singh RP, Agarwal R (2006). Natural flavonoids targeting deregulated cell cycle progression in cancer cells. Curr Drug Targets.

[9] Birt DF, Hendrich S, Wang W (2001). Dietary agents in cancer prevention: flavonoids and isoflavonoids. Pharmacol Ther.

[10] Weng CJ, Yen GC (2012). Flavonoids, a ubiquitous dietary phenolic subclass, exert extensive in vitro anti-invasive and in vivo anti-metastatic activities. Cancer Metastasis Rev.

[11] Izumi T, Piskula MK, Osawa S, Obata A, Tobe K, Saito M (2000). Soy isoflavone aglycones are absorbed faster and in higher amounts than their glucosides in humans. Nutr.

[12] Choi EJ, Kim T, Lee MS (2007). Pro-apoptotic effect and cytotoxicity of genistein and genistin in human ovarian cancer SK-OV-3 cells. Life Sci.

[13] Xiao JX, Huang GQ, Geng X, Qiu HW (2011). Soy-derived isoflavones inhibit HeLa cell growth by inducing apoptosis. Plant Foods Hum Nutr.

[14] Dong X, Xu W, Sikes RA, Wu C (2013). Combination of low dose of genistein and daidzein has synergistic preventive effects on isogenic human prostate cancer cells when compared with individual soy isoflavone. Food Chem.

[15] Hsu A, Bray TM, Helferich WG, Doerge DR, Ho E (2010). Differential effects of whole soy extract and soy isoflavones on apoptosis in prostate cancer cells. Exp Biol Med.

[16] Klein A, He X, Roche M, Mallett A, Duska L, Supko JG (2006). Prolonged stabilization of platinum-resistant ovarian cancer in a single patient consuming a fermented soy therapy. Gynecol Oncol.

[17] Leloup L, Wells A (2011). Calpains as potential anti-cancer targets. Expert Opin Ther Targets.

[18] Jánossy J, Ubezio P, Apáti A, Magócsi M, Tompa P, Friedrich P (2004). Calpain as a multi-site regulator of cell cycle. Biochem Pharmacol.

[19] Nair V, Fams MS (2004). Soy and cancer survivors: Dietary supplementation with fermented soy nutraceutical, Haelan 951 in patients who survived terminal cancers. Townsend letter for doctors & patients.

[20] Wang X, Clubbs EA, Bomser JA (2006). Genistein modulates prostate epithelial cell proliferation via estrogen- and extracellular signal-regulated kinase-dependent pathways. J Nutr Biochem.

[21] Hsieh CY, Santell RC, Haslam SZ, Helferich WG (1998). Estrogenic effects of genistein on the growth of estrogen receptor-positive human breast cancer (MCF-7) cells in vitro and in vivo. Cancer Res.

[22] Murata M, Midorikawa K, Koh M, Umezawa K, Kawanishi S (2004). Genistein and daidzein induce cell proliferation and their metabolites cause oxidative DNA damage in relation to isoflavone-induced cancer of estrogen-sensitive organs. Biochemistry.

[23] Wang C, Kurzer MS (1997). Phytoestrogen concentration determines effects on DNA synthesis in human breast cancer cells. Nutr Cancer.

[24] Hollande E, Fanjul M, Houti N, Faye JC, Courriere P (1998). Expression of estrogen receptors during growth of human pancreatic adenocarcinoma cells (Capan-1)-relationship with differentiation. In Vitro Cell Dev Biol Anim.

[25] Haczynski J, Tarkowski R, Jarzabek K, Slomczynska M, Wolczynski S, Magoffin DA (2002). Human cultured skin fibroblasts express estrogen receptor alpha and beta. Int J Mol Med.

[26] Choi EJ, Lee BH (2004). Evidence for genistein mediated cytotoxicity and apoptosis in rat brain. Life Sci.

[27] Jin Y, Wu H, Cohen EM, Wei J, Jin H, Prentice H (2007). Genistein and daidzein induce neurotoxicity at high concentrations in primary rat neuronal cultures. J Biomed Sci.

[28] Büchler P, Gukovskaya AS, Mouria M, Büchler MC, Büchler MW, Friess H (2003). Prevention of metastatic pancreatic cancer growth in vivo by induction of apoptosis with genistein, a naturally occurring isoflavonoid. Pancreas.

[29] Choi EJ, Kim GH (2014). The antioxidant activity of daidzein metabolites, O‑desmethylangolensin and equol, in HepG2 cells. Mol Med Rep.

[30] McCabe MJ, Orrenius S (1993). Genistein induces apoptosis in immature human thymocytes by inhibiting topoisomerase-II. Biochem Biophys Res Commun.

[31] Ye R, Bodero A, Zhou BB, Khanna KK, Lavin MF, Lees-Miller SP (2001). The plant isoflavenoid genistein activates p53 and Chk2 in an ATM-dependent manner. J Biol Chem.

[32] Vousden KH (2006). Outcomes of p53 activation-spoilt for choice. J Cell Sci.

[33] Polkowski K, Mazurek AP (2000). Biological properties of genistein. A review of in vitro and in vivo data. Acta Pol Pharm.

[34] Yakovlev AG, Di Giovanni S, Wang G, Liu W, Stoica B, Faden AI (2004). BOK and NOXA are essential mediators of p53-dependent apoptosis. J Biol Chem.

[35] Gonen H, Shkedy D, Barnoy S, Kosower NS, Ciechanover A (1997). On the involvement of calpains in the degradation of the tumor suppressor protein p53. FEBS Lett.

[36] Guan N, Korukonda R, Hurh E, Schmittgen TD, Donkor IO, Dalton JT (2006). Apoptosis induced by novel aldehyde calpain inhibitors in human tumor cell lines. Int J Oncol.

[37] Delmas C, Aragou N, Poussard S, Cottin P, Darbon JM, Manenti S (2003). MAP kinase-dependent degradation of p27Kip1 by calpains in choroidal melanoma cells. Requirement of p27Kip1 nuclear export. J Biol Chem.

[38] Zhu W, Murtha PE, Young CY (1995). Calpain inhibitor-induced apoptosis in human prostate adenocarcinoma cells. Biochem Biophys Res Commun.

[39] Atencio IA, Ramachandra M, Shabram P, Demers GW (2000). Calpain inhibitor 1 activates p53-dependent apoptosis in tumor cell lines. Cell Growth Differ.

[40] Łopatniuk P, Witkowski JM (2011). Conventional calpains and programmed cell death. Acta Biochim Pol.

[41] Chua BT, Guo K, Li P (2000). Direct cleavage by the calcium-activated protease calpain can lead to inactivation of caspases. J Biol Chem.

[42] Donato R, Cannon BR, Sorci G, Riuzzi F, Hsu K, Weber DJ (2013). Functions of S100 proteins. Curr Mol Med.

[43] Chen H, Xu C, Jin Q, Liu Z (2014). S100 protein family in human cancer. Am J Cancer Res.

[44] Tabata T, Tsukamoto N, Fooladi AA, Yamanaka S, Furukawa T, Ishida M (2009). RNA interference targeting against S100A4 suppresses cell growth and motility and induces apoptosis in human pancreatic cancer cells. Biochem Biophys Res Commun.

[45] Che P, Yang Y, Han X, Hu M, Sellers JC, Londono-Joshi AI (2015). S100A4 promotes pancreatic cancer progression through a dual signaling pathway mediated by Src and focal adhesion kinase. Sci Rep.

[46] Tamaki Y, Iwanaga Y, Niizuma S, Kawashima T, Kato T, Inuzuka Y (2013). Metastasis-associated protein, S100A4 mediates cardiac fibrosis potentially through the modulation of p53 in cardiac fibroblasts. J Mol Cell Cardiol.

